# Scientific Items

**Published:** 1886-07-20

**Authors:** 


### SCIENTIFIC ITEMS.

**The New Neubla in the P eiades.**—In the article describing a sun-spot, in the last number of the *Science News*, reference was made to a nebula existing in the group of stars known as the Pleiades, in the constellation of Taurus. It was first discovered by the Henry brothers, at the Paris Observatory, on the 16th of November last, when it appeared on a photographic plate on which they had been taking stellar photographs. Although it has been photographed several times, its light is so feeble that it could not be seen by direct telescopic observation; and only the surface of the photographic plate was sensitive enough to be affected by its feeble radiations. Quite recently, however, the nebula has been observed in the great thirty-inch refracting telescope of Pulkowa, Russia, the largest in the world, and the discovery is thus confirmed.

The discovery of this nebula is a most interesting and important occurrence, as showing the value of the photographic method in cases where the celestial bodies are too faint to be perceived by the human eye. Astronomers are at present paying great attention to this method; and although it is not a new discovery, as astronomical photographs have been taken for many years, still the many improvements recently introduced, and more especially the extreme sensitiveness of the dry plates now in use, have greatly increased the value of the process; and before long we may expect to hear of many other discoveries as brilliant as that of the brothers Henry.—*Popular Science News.*

**Investigating Ghosts.**—A committee has been appointed to investigate ghosts. The committee, as we have stated, undertake this investigation without preconceived prejudices. Being very bright people, they cannot help having their own opinions, and it would be safe to say that the majority are without belief in such appearances; but they wish simply to hear and examine the facts, and are pledged to draw from them such conclusions as are warranted by the evidence. They invite the co-operation of persons simillarly earnest and unprejudiced. Col. T. W. Higginson, Cambridge, Mass., is one of the committee. A limited time will also be devoted to the personal examination of houses in the neighborhood of Boston which are reported to be haunted.

In Philadelphia, an analogous though less open investigation is now in progress. Some years ago, Mr. Seybert, the donor of the new bell for Independence Hall, was the victim of severe imposition at the hands of several over-thrifty "mediums." At his death he left a sum of money to the University of Pennsylvania on the condition that it should thoroughly investigate the claims of spiritualism, and make the results public. The committee intrusted with the task has been collecting testimony on this point for a number of months, but no results, we believe, have yet been publishee. It is to be hoped that their labor will shortly be completed and their conclusions made public.—*Scientific American.*

**Fishing with Dynamite.**—By special invitation we were permitted to witness a novel experiment one afternoon recently, which was intended to test the efficacy of dynamite bombs in the capture of fish in deep water. The objective point was found to be a hole about twenty-five feet deep in the upper end of the bight, where the fish are known to congregate in large numbers. Arriving at the spot, a cartridge about six inches long, charged with dynamite, to which had been attached a heavy piece of iron in order to make it go to the bottom, was thrown in the water. A suspense of a few seconds ensued, and then a faint report like the discharge of a small pistol was heard, the water became agitated and was raised about two feet, and immediately thereafter, within a radius of about sixty feet, the fish were strewn in all directions. A scene of the wildest excitement followed. Scoop nets were brought into speedy use, and over 1,000 fish of different varieties, from the large gray snapper, over three feet in length, to the small but succulent sailor's choice, were secured.—*Key West Democrat.*

**A Physician's Buggy Case** has been patented by Mr. Joseph J. Stevens, of Coalesburg, Mo. This invention consists mainly in the manner of combining two opposite medicine or instrument boxes, and attaching to them a single lid, making a case convenient to carry in a buggy or in the hand, when the medicines and instruments will be easily accessible, and which is waterproof.—*Scientific American.*

**What a Frenchman can do with a Hair.**—Of Gen. von Manteuffel, the late German military governor of conquered Alsace, who hated all that was French, it is said that he once, at a public dinner, engaged in a dispute with a French diplomat, who maintained the superiority of the French workmen over the artisans of all other nations. "A thing so ugly does not exist that the skill and genius of a Frenchman cannot make of it a thing of beauty," he said. Angered by the contradiction, the old soldier pulled a hair from his bristly gray mustache, and, handing it to the Frenchman, said curtly, "Let him make a thing of beauty out of that, then, and prove your claim." The Frenchman took the hair, and sent it in a letter to a well-known Parisian jeweller, with a statement of the case and an appeal to his patriotic pride, giving him no limit of expense in executing the order. A week later the mail from Paris brought a neat little box for the General. In it was a handsome scarf-pin made like a Prussian eagle, that held in its talons a stiff gray bristle, from either end of which dangled a tiny golden ball. One was inscribed "Alsace," and the other "Lorraine;" and on the eagle's perch were the words, "You hold them, but by a hair."—*Popular Science News.*

**To Destroy Wood Worms.**—These creatures can be destroyed in books and woodwork by benzine. Books are locked up in a cupboard with a saucer of benzine. The insects, as well as their larvæ and eggs, soon die off. Furniture and carvings are similarly placed in a room with a dish of benzine, and kept closed up for the complete destruction of the insects varying according to the thickness of the wood.—*Ibid.*

